# Control of the wrinkle structure on surface-reformed poly(dimethylsiloxane) via ion-beam bombardment

**DOI:** 10.1038/srep12356

**Published:** 2015-07-21

**Authors:** Hong-Gyu Park, Hae-Chang Jeong, Yoon Ho Jung, Dae-Shik Seo

**Affiliations:** 1 Department of Electrical and Electronic Engineering, Yonsei University, 50 Yonsei-ro, Seodaemun-gu, Seoul 120-749, Republic of Korea

## Abstract

We investigated the surface reformation of poly(dimethylsiloxane) (PDMS) elastomers by means of ion beam bombardment for fabricating wrinkle structures. Oxidation on the PDMS surface formed a silica-like outer layer that interacted with the inner PDMS layer, leading to the formation of wrinkle structures that minimized the combined bending energy of the outer layer and stretching energy of the inner layer. In addition, we controlled the amplitude and period of the wrinkle structures by adjusting the PDMS annealing temperature. As the PDMS annealing temperature was increased, the amplitude and period of the wrinkles formed by IB irradiation changed from 604.35 to 69.01 nm and from 3.07 to 0.80 μm, respectively.

Nanostructures and microstructures have traditionally been produced by means of photolithographic, printing, embossing or writing techniques^1^,^2^,^3^,^4^,^5^. However, these methods have relatively high costs and limited throughput in the production of customized features. Alternative spontaneous wrinkling fabrication methods have therefore attracted considerable attention in recent decades^6^,^7^ owing to their simplicity. These methods, which include plasma activation^8^, UV/ozone (UVO) treatment^9^,^10^, laser excitation^11^, and ion-beam (IB) treatment^12^, all entail surface reformation of a polymer layer, whereupon spontaneous wrinkling occurs that minimizes the combined bending energy of the outer layer and stretching energy of the inner layer. Moreover, control of the spontaneously formed wrinkle structures can enhance the mechanical^13^,^14^,^15^, electrical^16^ and optical^17^ properties of materials. Consequently, research efforts have been increasing in the study of topological wrinkle structures at the nanometre and micrometre scales. The findings can be applied to various systems, including stretchable electronics^18^, microlens arrays^19^, tunable surface adhesion^20^, alignment of nematic liquid crystals^15^,^21^ and tunable optics^9^,^22^,^23^.

There are many candidate materials for the fabrication of wrinkle structures^24^,^25^,^26^. In particular, poly (dimethylsiloxane) (PDMS) has attracted attention because of its versatility and potential for widespread application^27^,^28^,^29^,^30^,^31^. To induce wrinkling of PDMS, its surface is commonly subjected to UVO treatment or IB irradiation. Many research groups have additionally adopted various methods by adding dependent factor and have demonstrated the theoretical reason. Representatively, K. Efimenko *et al*. investigated the formation of hierarchical wrinkle structures by means of stretching PDMS substrates simultaneously during UVO irradiation treatment^32^. N. Stoop *et al*. demonstrated curvature-induced wrinkle patterning^33^. E. P. Chan and A.J. Crosby manipulated elastic moduli to achieve variations in local stress, thereby obtaining oriented wrinkle patterns^34^. The various methods available for the fabrication of wrinkle structures on PDMS demonstrate its versatility as a material. This versatility is expected to allow considerable flexibility in future applications.

Herein we present a simple method for fabricating wrinkles on PDMS. The method includes IB treatment, and the PDMS annealing temperature is a dependent factor. Wrinkle structures were fabricated on IB-irradiated PDMS surfaces, and their presence was confirmed by using physicochemical analysis. The mechanical properties of the two perfectly adhered layers, the skin layer and the substrate, were measured to characterise the controllable wrinkle structures.

## Results and Discussion

**Figure 1:**
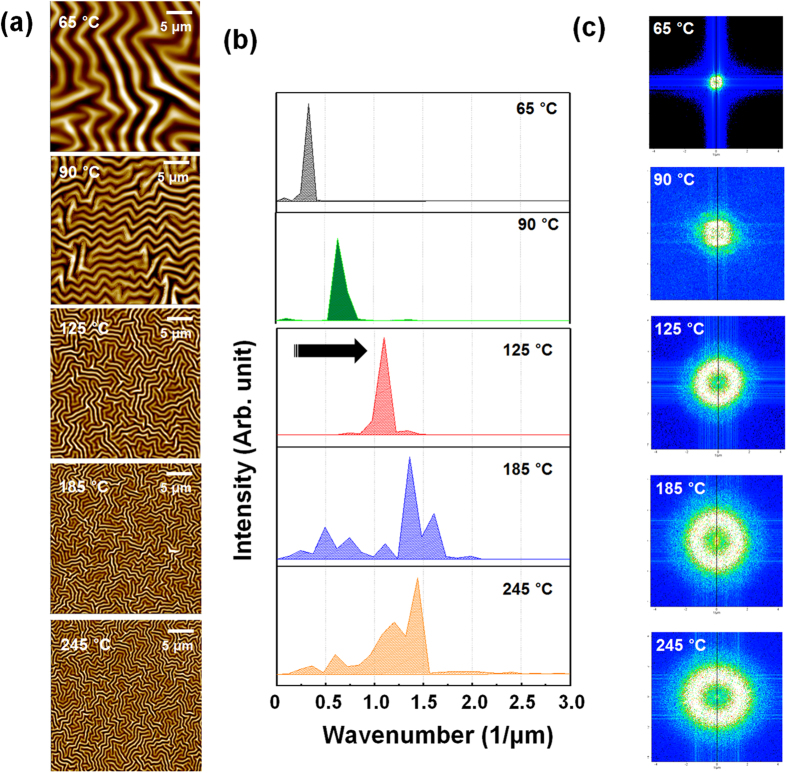
(**a**) AFM images, (**b**) wavenumber distributions and (**c**) 2D FFT patterns of wrinkle structures formed on PDMS films annealed at various temperatures. Increased PDMS annealing temperature led to decreased periods of the wrinkle structures that formed during subsequent IB irradiation.

Figure 1 shows atomic force microscopy (AFM) images in which the morphologies of wrinkled structures on PDMS surfaces are visible, and corresponding analyses of the wrinkles’ periods. As shown in Fig. 1a, the wrinkle structures became smaller as the annealing temperature was increased. The wrinkles’ wavenumbers (i.e., the inverse of the wrinkles’ periods) tended to increase as the annealing temperature was increased (Fig. 1b). The periods of the wrinkle structures were measured by using AFM 2D FFT power spectrum, as shown in Fig. 1c. The 2D power spectrum represents the power (including amplitude information) distribution of wrinkle pattern over spatial frequency domain converted from the spatial domain. The white point (the highest peak in the power spectrum) distributions indicate the periodicity in the surface morphology. The size of this white circular distribution increased with PDMS annealing temperature, indicating that the wrinkle wavelength decreased as the annealing temperature increased, consistent with the results of the wavenumber analysis.

**Figure 2:**
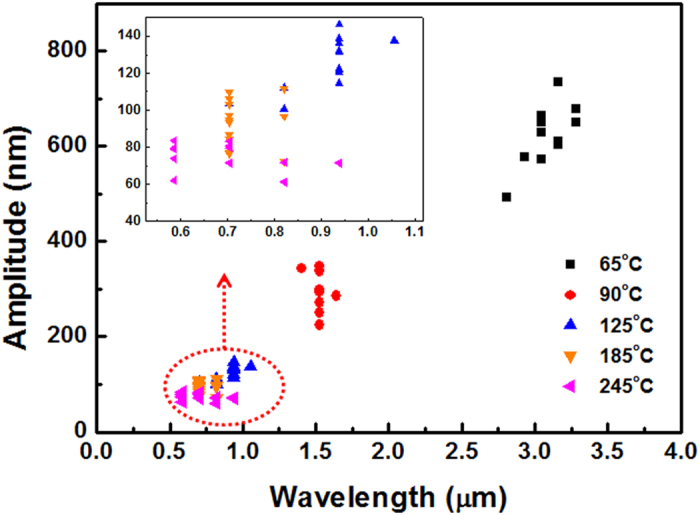
Amplitudes and wavelengths of wrinkle structures that formed on PDMS samples annealed at various temperatures.

Figure 2 shows the relationships between the periods and amplitudes of the wrinkle structures, based on AFM line profiles. As the annealing temperature was increased, the wavelengths of the PDMS wrinkle structures decreased from 3.07 to 0.80 μm, and their amplitudes decreased from 604.35 to 69.01 nm. Table 1 summarizes the average wavelengths and amplitudes of the PDMS wrinkle structures. Among the annealing temperatures studied, annealing at 65 °C produced the largest periods and amplitudes.

**Figure 3:**
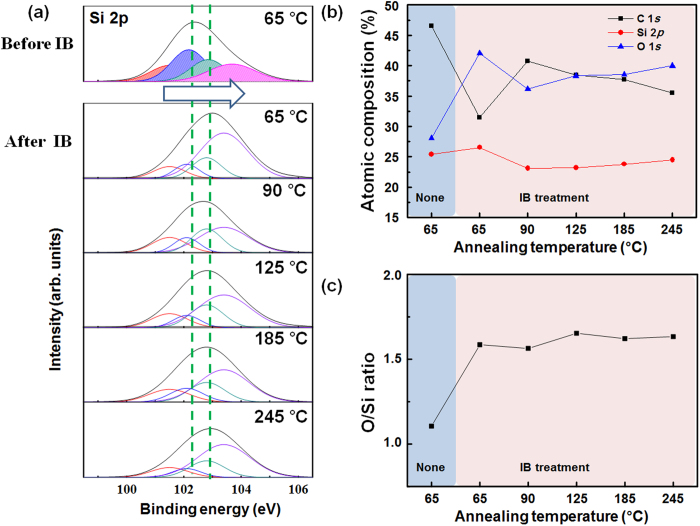
(**a**) Si 2p peak analysis of a PDMA sample annealed at 65 °C, and of PDMA samples annealed at various temperatures and subsequently subjected to IB irradiation. (**b**) Atomic concentrations of Si, O and C versus PDMA annealing temperature. (**c**) O/Si atomic concentration ratio versus PDMA annealing temperature.

It has been demonstrated that the IB irradiation modified the chemical composition of the surface into oxidized state^12^. To observe the effects of IB irradiation as a function of annealing temperature we chemically analysed PDMS samples by means of X-ray photoelectron spectroscopy (XPS). We analysed the binding energy of the Si 2p peak in the PDMS (Fig. 3a), which is related to Si and O bonding. There were four component subpeaks representing Si–O bonding: (–O)_1_: [(CH_3_)_3_SiO_1/2_], (–O)_2_: [(CH_3_)_2_SiO_2/2_], (–O)_3_: [(CH_3_)SiO_3/2_], and (–O)_4_: [SiO_4/2_]. The (–O)_1_ peak is centred at 101.5 eV, (–O)_2_ is centred at 102.1 eV, (–O)_3_ is centred at 102.8 eV, and (–O)_4_ is centred at 103.4 eV^35^. The rightward shift of the Si 2p peak indicated that IB irradiation transformed atoms at the PDMS surface into higher oxidation states. However, changes in annealing temperature did not greatly shift the Si 2p peak within after IB irradiation.

Figure 3b shows the surface atomic concentrations of Si, O and C in samples annealed at various temperatures. After IB irradiation, the concentration of carbon was drastically decreased and the concentration of oxygen was increased. Moreover, the composition of irradiated samples varied when different annealing temperatures were used. Annealing at 90 °C yielded a sample with considerably higher carbon content than annealing at 65 °C yielded; further increases in annealing temperature gradually decreased the observed carbon content. During an experiment in atmospheric conditions, samples exposed to atmosphere could acquire detectable adventitious contamination^36^,^37^. The large variations in carbon concentration among the samples annealed at different temperatures might be attributed to this adventitious contamination. We disregarded the carbon content and analysed the O/Si atomic concentration ratio because the Si atoms form the backbone of the PDMS polymer strand (Fig. 3c). These analyses revealed a variation of the Si 2p peak, which is related to oxidization. Before IB irradiation, the O/Si ratio of was about 1.1, close to that of pure PDMS. After IB irradiation, the O/Si ratios averaged 1.611, similar to previously reported results^38^. The stoichiometric compositions of the IB-irradiated PDMS samples were similar to that of SiO_x>3/2_, that is, silica-like layers (see Supplementary Fig. S1 online). However, the O/Si ratios were almost identical among all IB-irradiated samples, regardless of the annealing temperature, strongly suggesting that the formation of the silica-like skin layer has no connection with the annealing temperature. This result was consistent with the result that the Si 2p peak varied little with annealing temperature among the IB-irradiated samples (Fig. 3a). The samples’ surface oxidation was almost identical. The formation of a silica-like skin layer by means of IB irradiation is unaffected by annealing temperature and the IB-irradiated samples might have a constant stiffness regardless of annealing temperature.

To confirm the aforementioned conclusions, based on XPS results, that there was no relationship between annealing temperature and surface modification, we conducted an indentation experiment to acquire AFM force–distance curves for the IB-irradiated samples annealed at different temperatures; the gradients of these curves in the contact and compression regimes were nearly identical (see Supplementary Fig. S2 online). Sneddon’s relationship^39^ (F = 2 E r h/(1 − m^2^), where F is load, h is penetration depth, E is modulus, r is radius of contact area and m is Poisson’s ratio, assumed to be nearly 0.5^40^) was used to compute the Young’s moduli for all samples; these moduli ranged from 21 to 23 MPa, similar to previously reported results^41^. This indicates that annealing temperature has little effect on the stiffness of a skin layer modified by IB, consistent with the XPS analysis.

**Figure 4:**
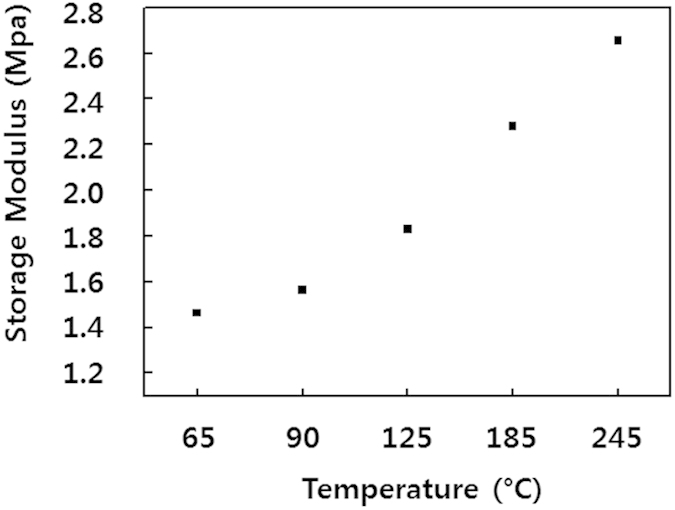
Storage modulus versus annealing temperature of PDMS substrates, measured by means of DMA.

Despite the fact that wrinkle wavelength and amplitude varied with annealing temperature, variations in the annealing temperature did not cause any noticeable mechanical or chemical changes in the skin layer (whereas the variation in IB intensity modified the chemical composition of the surface, thereby changing the wrinkle pattern (see Supplementary Fig. S3 and Fig. S4 online). Accordingly, to determine the cause of the variations in wrinkle size, we focused on analysing the mechanical properties of the PDMS substrates under the skin layer. We carried out dynamic mechanical analysis (DMA) to determine the variation in storage moduli, which are also called Young’s moduli (Fig. 4). The storage moduli of PDMS substrates annealed at 65 and 185 °C were 1.44 and 2.28 MPa, respectively. This increasing trend is likely to be closely related to the observed differences in wrinkle properties mentioned above, given that the skin layers were mechanically and chemically similar at different annealing temperature.

**Figure 5:**
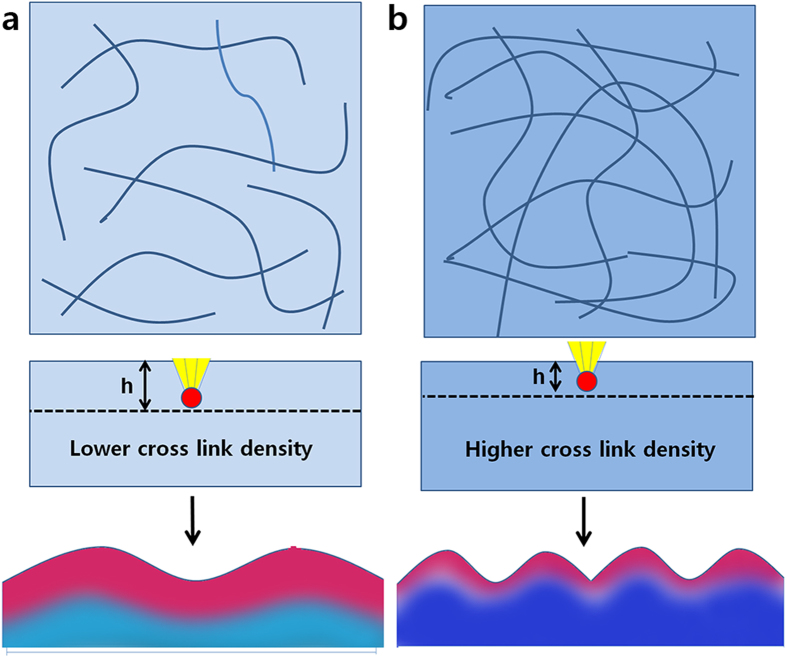
Schematic diagram illustrating the proposed mechanism whereby shorter wrinkle wavelengths formed in PDMA samples annealed at higher temperatures prior to IB irradiation. (**a**) PDMS annealed at low temperature, yielding low Young’s modulus of the PDMS substrate; (**b**) PDMS annealed at high temperature, yielding high Young’s modulus of the PDMS substrate.

We propose a possible mechanism explaining the reduction in wrinkle wavelength with increased annealing temperature, illustrated in Fig. 5. The ion-beam method is carried out by using an ion accelerator to accelerate reactive ions, thereby imparting them with sufficient energy to penetrate into the bulk^42^,^43^,^44^. The reactive ions modify the chemical composition of the surface as deeply as their penetration depth^21^,^45^. In the present work, irradiated PDMS was transformed into silica-like material, forming a stiff skin layer detectable by means of XPS. Theoretically, the formation of a stiff skin layer broke the material’s symmetry, thereby inducing surface undulations, i.e., wrinkle patterns. The inner layer is characterized by stretching energy; increased stretching energy causes the wrinkle wavelength to increase^46^. In contrast, the outer layer is characterized by bending energy; increased bending energy causes the wrinkle wavelength to decrease^46^. These competing effects can be expressed by the simple expression given as equation (1); M.-W Moon *et al*. demonstrated that this equation describes well the wrinkle wavelengths of IB-irradiated PDMS^45^.


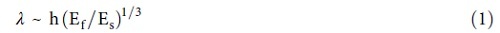


Here, E_f_ and E_s_ are the Young’s moduli of the silica-like skin layer and the substrate, respectively; λ is the wrinkle wavelength and h is the thickness of the silica-like skin layer. As described by equation (1), the wrinkle wavelength is proportional to the thickness of skin layer and to the cube root of the ratio between the moduli of the skin layer and the substrate. By using the aforementioned results of indentation experiments and DMA, we can derive the relation between the wavelength term (λ) and the modulus term ((E_f_/E_s_)^1/3^). The skin layer moduli were nearly constant with varying annealing temperatures, and the substrate modulus increased linearly by about 1.7 times as the annealing temperature was increased from 60 °C to 245 °C. The cube root of this factor of 1.7 is approximately 1.19 and the reciprocal of 1.19 is 0.84. According to the equation (1), the 84% reduction of the cube root ratio of elastic modulus between skin layer and substrates was insufficient to explain the observed half reduction of wrinkle wavelength for the increase in annealing temperature; accordingly, we concluded that the observed reduction of wrinkle wavelength was also attributed to decreasing skin layer thickness (h) as the annealing temperature was increased.

In most cases, the thickness of the skin layer is a dominant factor^25^. It has been demonstrated that, when the surface of PDMS is transformed to a silica-like layer by oxygen or UVO treatment, the depth of treatment can be controlled by the density of cross-linking in the PDMS material^41^,^47^. In the present work, the PDMS substrates modulus was found to increase with increasing annealing temperature; this indicates that the application of higher annealing temperatures increased the degree of polymer crosslinking. The increased crosslinking was accompanied by a decrease in the number of polymer chains, leading to increased rigidity and decreased free volume in the polymer structure. Such changes to the structure of PDMS annealed at high temperature might restrict the local movement of reactive ions during subsequent ion beam irradiation treatment^47^. Such restricted movement would reduce the penetration depth of the reactive ions and lead to the formation of thinner silica-like skin layers, thereby reducing the wrinkle wavelength.

In summary, we fabricated wrinkle PDMS surfaces with wrinkles of various sizes by means of annealing at various temperatures followed by IB irradiation, and we confirmed the formation of these structures by means of various analyses including AFM, XPS and DMA. When the PDMS layers were annealed at high temperatures, their surface stiffness was not changed (as confirmed by XPS analysis and AFM force–distance measurements), but the storage modulus of the substrate increased. The increased stiffness of PDMS substrates annealed at higher temperature might restrict the ion penetration depth, thereby controlling the wrinkle wavelength.

## Methods

### Materials & preparation

PDMS precursor mixtures were prepared by combining Sylgard 184 base and hardener (Sylgard 184, Dow Corning Corporation, USA) in the ratio of 10:1 (by weight) by means of mixing in a beaker for several minutes with a spatula. The prepared mixtures were then placed in desiccators and degassed under vacuum until no bubbles remained in the bulk of the mixtures. Glass substrates were prepared for coating by means of a standard cleaning method comprising sequential ultrasonication in trichloroethane, acetone, methanol and deionized water for 10 min each, following by drying with N_2_ gas. The precursor mixtures were spin coated onto 22 × 30 mm glass substrates for 30 s at 3000 rpm. Next, the PDMS coatings were annealed by heating them on hotplates for 120 min. Various annealing temperatures were used: 65, 90, 125, 185 and 245 °C. This procedure yielded PDMS films 20 μm thick.

### Ion beam (IB) irradiation

To fabricate wrinkles, PDMS surfaces prepared by different annealing temperature were exposed to Ar^+^ IB plasma at doses of 10^14^–10^15^ ions/cm^2^ of energy 2400 eV for 2 min by using a DuoPIGatron-type IB system. The current density in the beam of positively charged particles was 1.1 mA/cm^2^, and the plasma ion density was approximately 10^11^ cm^−3^. An experiment using the variable of IB intensity was performed by using samples all annealed at 90 °C; the intensities studied were 300, 800, 1500 and 2400 eV.

### AFM analysis of wrinkle structure

The morphologies of wrinkled surfaces were observed by means of AFM (XE-BIO, Park Systems). The acquired AFM images were inspected to locate one line profile in each image that would show the characteristic amplitudes and wavelengths of the wrinkles. Additionally, the AFM images were subjected to 2D FFT analysis to characterise the wrinkles’ wavelengths and alignment patterns.

### Chemical composition analysis

Chemical bonding states of PDMS film surfaces were analysed by using an XPS instrument (K-alpha, Thermo VG, UK) equipped with a monochromated Al X-ray source (Al Kα line: 1486.6 eV) and operated at the power of 12 kV and 3 mA.

### Measurement of skin layer using the force-distance approach

To determine the elastic modulus of the IB-irradiated skin layer in samples annealed at various temperatures, the samples were subjected to indentation experiments carried out by using the AFM force–distance approach, using the compression depth of 80 nm. The results were used to calculate the moduli by means of Sneddon’s relationship. Pyramidal silicone tips were used that had the contact area radius of 6 nm.

### DMA to investigate the modulus of the substrate under the skin layer

DMA of PDMA samples annealed at various temperatures was conducted by using a dynamic mechanical analyser (TA; DMA Q800) according to ASTM D4065 (Standard Practice for Plastics: Dynamic Mechanical Properties: Determination and Report of Procedures). PDMS sheets of dimensions 20 mm × 50 mm × 1 mm were loaded into a film tensile clamp, and the analyser was operated in single-frequency scanning mode at 1 Hz, using the heating rate of 10.0 °C/min over the temperature range from 50 to 250 °C.

## Additional Information

**How to cite this article**: Park, H.-G. *et al*. Control of the wrinkle structure on surface-reformed poly(dimethylsiloxane) via ion-beam bombardment. *Sci. Rep*. **5**, 12356; doi: 10.1038/srep12356 (2015).

## Supplementary Material

Supplementary Information

## Figures and Tables

**Table 1:** Average periods and amplitudes of the wrinkle structures formed at various PDMS annealing temperatures.

**Annealing temperature (°C)**	**65**	**90**	**125**	**185**	**245**
Period (μm)	3.07	1.52	1.04	0.88	0.80
Amplitude (nm)	604.35	285.68	119.81	92.27	69.01
Aspect ratio	0.20	0.18	0.12	0.11	0.09
