# Supplementary material for: Control of the wrinkle structure on surface-reformed poly(dimethylsiloxane) via ion-beam bombardment
Source: Sci Rep. 2015 Jul 21;5:12356. doi: 10.1038/srep12356 (PMC4508831; doi:10.1038/srep12356)
Supplement: Supplementary Information [file srep12356-s1.doc]

**SUPPLEMENTARY INFORMATION**

**Control of the wrinkle structure on surface-reformed poly(dimethylsiloxane) via ion-beam bombardment**

Hong-Gyu Park^1,2^, Hae-Chang Jeong^1,2^, Yoon Ho Jung^1^, and Dae-Shik Seo^1,*^

^1^Department of Electrical and Electronic Engineering, Yonsei University, 50 Yonsei-ro, Seodaemun-gu, Seoul 120-749, Republic of Korea

^1^Department of Electrical and Electronic Engineering, Yonsei University, 50 Yonsei-ro, Seodaemun-gu, Seoul 120-749, Republic of Korea

^2^ Authors contributed equally.


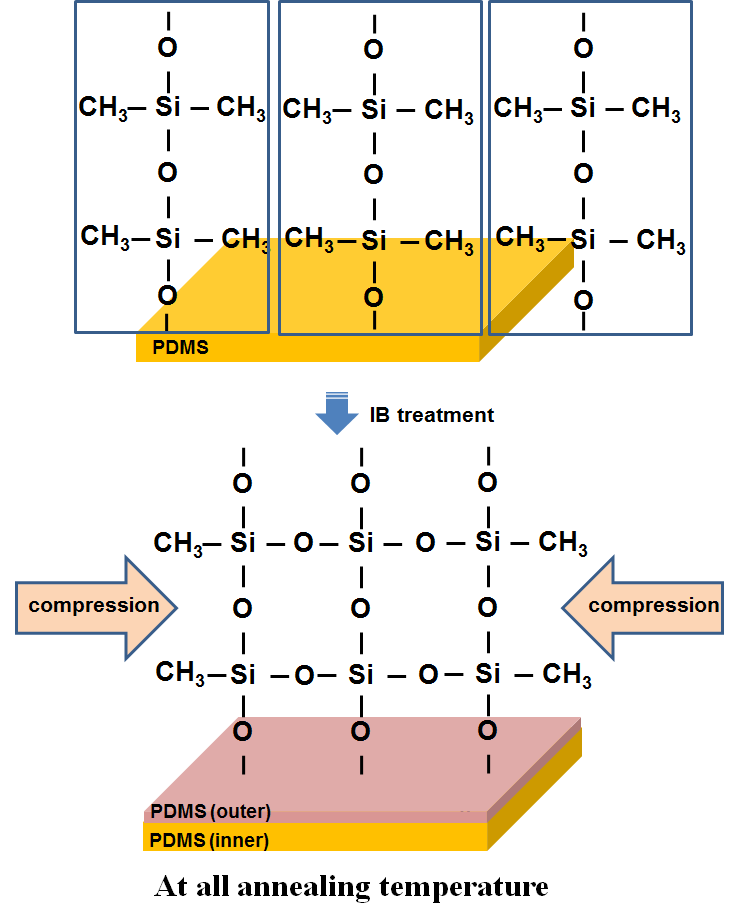


Figure S1 Polymer structures of the PDMS surface before and after IB irradiation, presumed based on the measurements of O/Si stoichiometry.

Figure S1 shows possible polymer structures before and after IB irradiation, based on the assumption that the polymer structure was identical regardless of the annealing temperature; these structures are consistent with the aforementioned stoichiometric compositions that were determined by means of the analyses depicted in Figs. 3a and 3c. We believe that most methyl groups were eliminated from each polymer strand by means of the IB irradiation treatment, and that new O–Si–O interchain bonds were subsequently formed, thereby changing the surface PDMS into a stiff silica-like skin layer of SiO_x>3/2_ .


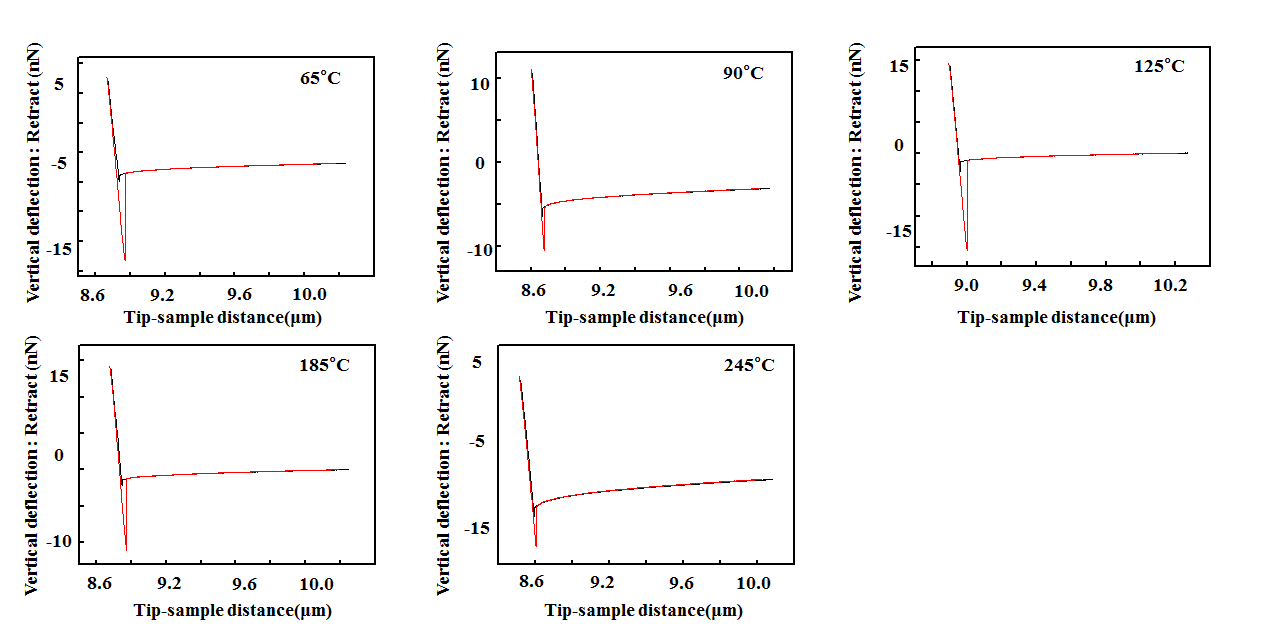


**Figure S2. Force–distance plots acquired during indentation tests of samples annealed at various temperatures.**

The relationship between elastic modulus and annealing temperature was investigated by means of indentation experiments using an AFM force–distance approach. PDMS samples annealed at various temperatures and treated by IB irradiation were subjected to indentation measurements using 80 nm of compression, and the corresponding moduli were calculated by using Sneddon’s relationship (F = 2 E r h / (1−m^2^), where F is load, h is penetration depth, E is modulus, r is radius of contact area and m is Poisson’s ratio, assumed to be nearly 0.5 ^3^). Pyramidal silicone tips were used that had the contact area radius of 6 nm.

In the resulting plots of elastic modulus versus annealing temperature, the gradients in contact and compression modes were nearly the same regardless of annealing temperature (Fig. S2). The gradient is the ratio between load (F) and penetration depth (h); the gradient values extracted from indentation plots for samples annealed at 65, 90, 125, 185 and 245 °C were 0.37348, 0.36225, 0.38662, 0.34469 and 0.36615, respectively. The corresponding Young’s moduli were computed to be 23, 23, 23, 22 and 23 MPa. The computed values were therefore constant regardless of annealing temperature.


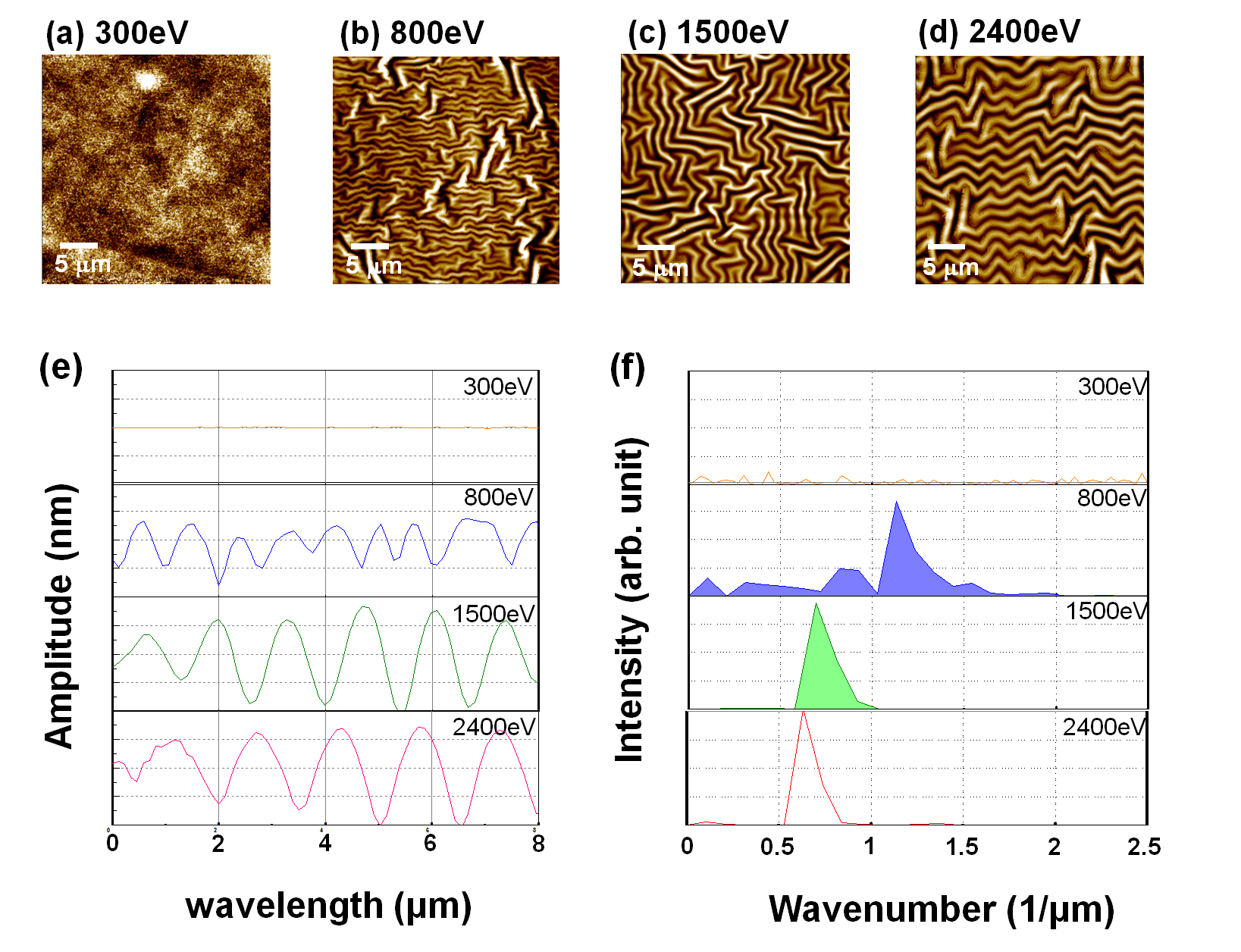


**Figure S3** (a–d) Wrinkle formation in PDMS treated by IB irradiation of various ion energies: (a) 300 eV, (b) 800 eV, (c) 1500 eV and (d) 2400 eV. Each image shows an area of 30 µm × 30 μm^2^. (e) Line profiles illustrating cross sections of wrinkled surfaces prepared using various IB energies; in each graph, the y-axis ranges from −200 to 200 nm. (f) Power spectra illustrating the distributions of wavenumbers, which are the reciprocals of the wavelengths.

Fig. S3 presents AFM images detailing the wrinkle morphologies of samples annealed at 90 °C and treated by means of IB irradiation using various IB energies ranging from 300 to 2400 eV. In PDMS wrinkle fabrication methods, typically the PDMS surface is transformed into a silica-like layer by IB irradiation; then the resulting differences between the silica-like layer and the underlying PDMS film lead to wrinkling. No wrinkles were observed when 300 eV IB irradiation treatment was used (Fig. S1a); evidently, this energy level was insufficient to form a silica-like layer on the PDMS surface. Wrinkles were formed for all treatment energies studied of 800 eV and greater, and the amplitude and wavelength increased with increases in the IB treatment energy. The small wrinkles formed by treatment at 800 eV had the average wavelength of 0.9 μm and average amplitude of 120 nm (Fig. S1b). Treatment at 1500 eV yielded wrinkles of average wavelength and amplitude 1.5 μm and 280 nm, respectively. Treatment at 2400 eV yielded wrinkles of average wavelength and amplitude 1.52 μm and 286 nm, respectively. The wrinkle wavelengths and amplitudes were very similar between the samples irradiated using the energies of 1500 and 2400 eV.


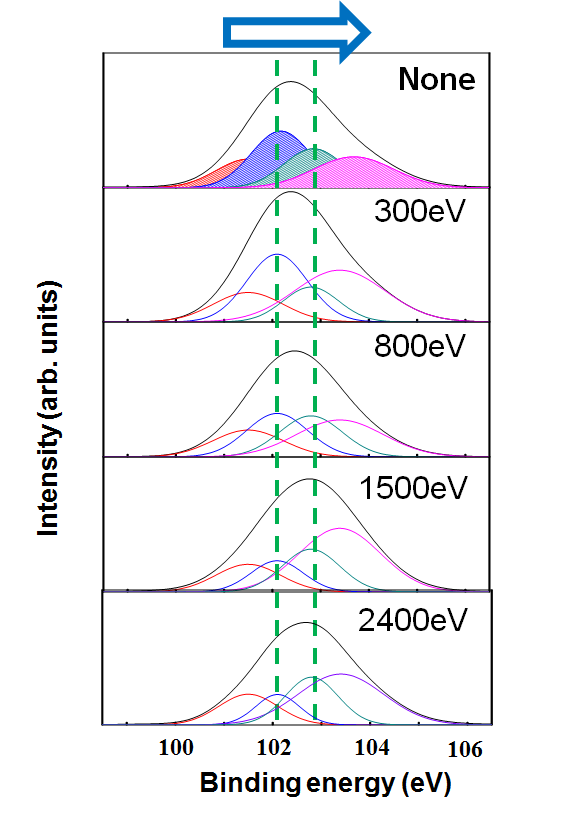


**Figure S4.** Si 2p core-level XPS spectra of PDMS surfaces treated using various IB energies.

PDMS, a silicone, is an organic polymer consisting of repeated Si(CH_3_)_2_O monomer units. To confirm the influence of the IB irradiation conditions, we conducted additional tests in which IB energy was the experimental variables. We analysed the binding energy of the resulting Si 2p peaks of the PDMS samples, which are related to Si and O bonding. The four component subpeaks, which represent Si–O bonding, are (–O)_1_: [(CH_3_)_3_SiO_1/2_], (–O)_2_: [(CH_3_)_2_SiO_2/2_], (–O)_3_: [(CH_3_)SiO_3/2_], and (–O)_4_: [SiO_4/2_]. The (–O)_1_ subpeak is centred at 101.5 eV, (–O)_2_ is centred at 102.1 eV, (–O)_3_ is centred at 102.8 eV, and (–O)_4_ is centred at 103.4 eV ^1^. Nonirradiated PDMS had relatively large (–O)_1_ and (–O)_2_ subpeaks; after IB irradiation, the (–O)_1_ and (–O)_2_ subpeaks were reduced in size and the (–O)_3_ and (–O)_4_ subpeaks were enlarged. When higher IB energy was applied, more oxygen atoms bonded with each Si atom, increasing the relative contributions of the (–O)_3_ and (–O)_4_ subpeaks. The Si 2p peak shifted rightward as the IB energy was increased; because higher binding energies represent atoms of higher positive oxidation state, this result indicated that as IB energy was increased, Si–C bonds were more likely to decompose, and more O atoms replaced the C atoms to form O–Si–O bonds. The ion beam energy of 300 eV appeared to be insufficient to form wrinkles, whereas energies of 900 eV and greater were observed to break the bonds and form wrinkles. If C atoms were not replaced by O atoms, the silica-like layer would not form and no wrinkles would be observed. This result is supported by the variations observed in subpeak composition related to the Si–O bond. IB irradiation transformed the PDMS to an oxidised state, and at higher IB energies, more oxygen atoms bonded with each Si atom, thereby forming stiffer skin layers and leading to the formation of wrinkle structures having long wavelengths. Little shift of the Si 2p peak was observed between the samples treated at 1500 and 2400 eV; this indicated that the 1500 eV IB energy was sufficient to fully oxidise the surface and form a silica-like layer of SiO_x_. The nearly identical wavelengths of wrinkles observed in samples treated at 1500 and 2400 eV could be attributed to the full oxidation of the skin layer in these samples.

**REFERENCES**

1 Kaul, A. D., Gangwal, A. and Wadhwa, S. S. Nanoscale measurements for computing Young’s modulus with atomic force microscope. *Curr. Sci.* **76,** 1561 (1999).

2 Kim, P., Abkarian, M., and Stone, H. A. Hierarchical folding of elastic membranes under biaxial compressive stress. *Nat. Mater.* **10**, 952 (2011).

3. Alexander, M. R. et al. An X-ray photoelectron spectroscopic investigation into the chemical structure of deposits formed from hexamethyldisiloxane/ oxygen plasmas. *J. Mater. Sci.* **31**, 1879 (1996).
